# Role of polycyclic aromatic hydrocarbons as a co-factor in human papillomavirus-mediated carcinogenesis

**DOI:** 10.1186/s12885-019-5347-4

**Published:** 2019-02-11

**Authors:** Chuqing Zhang, Yunjing Luo, Rugang Zhong, Priscilla T. Y. Law, Siaw Shi Boon, Zigui Chen, Chi-Hang Wong, Paul K. S. Chan

**Affiliations:** 1 Departments of Microbiology, The Chinese University of Hong Kong, Shatin, NT Hong Kong; 2 Beijing Key Laboratory of Environment and Viral Oncology, Beijing University of Technology, Beijing, People’s Republic of China; 3 Departments of Clinical Oncology, The Chinese University of Hong Kong, Prince of Wales Hospital, Shatin, New Territories, Hong Kong Special Administrative Region People’s Republic of China

**Keywords:** Polycyclic aromatic hydrocarbons, Human papillomavirus, Cancer, Cofactors, Pollution

## Abstract

**Background:**

Human papillomavirus (HPV) is an etiological agent of cervical cancer. Yet co-factors are believed to be involved in HPV-mediated carcinogenesis. Polycyclic aromatic hydrocarbons (PAHs) are considered as one of these co-factors. Epidemiologic studies have associated high PAH exposure with increased risk for cancer development. To date, many studies focus on benzo[a]pyrene, however, the role of other PAHs should not be neglected. This study aimed to compare the potential of different PAHs as a co-factor in HPV-mediated carcinogenesis, and to investigate the possible mechanisms involved.

**Methods:**

The effect of 17 PAHs on high-risk HPV (HPV16) were examined in this study. HPV16 E7 oncogene was expressed in primary cells extracted from baby rat kidney and treated with PAHs. The co-transforming ability of PAHs were measured by colony formation index according to the number and size of transformed colonies. Effects of PAHs on proliferation of HPV-null (C33A) and –infected (CaSki) were examined using CCK-8 assay. Wound healing assay and matrigel invasion chambers were used to investigate effects of PAHs on cell motility and invasivion of HPV-null (MCF7, C33A) and –infected (SiHa) cells.

**Results:**

Benzo[a]pyrene (BaP), dibenz[a,h]anthracene (DBA) and indeno[1,2,3-cd]pyrene (IDP) showed the greatest co-transforming potential in the baby rat kidney cell system. Short-term exposure to BaP, DBA, IDP and pyrene (PR) did not affect proliferation of C33A or CaSki cells, however, long-term exposure of these four PAHs led to dramatic increase in growth rate of CaSki cells by 120–140%. Besides, exposure of PAHs has an effect on cell motility and invasiveness of C33A and SiHa cells, but not for MCF7 cells. Exposure of BaP and DBA enhanced migration (1.26 to 1.40-fold) and invasion (1.68 to 1.94-fold) capacity of C33A cells. Intriguingly, exposure of all four types of PAHs boosted the migration (1.12 to 1.28-fold) and invasion (1.26 to 1.40-fold) capacity of SiHa cells.

**Conclusions:**

Our results indicate that exposure to PAHs can be a key co-factor in HPV-related cancer development. They could act on all three stages, namely initiation, promotion and progression. Further study is needed to unveil the mechanisms by which PAHs interact with HPV to cause malignancy.

## Background

Cervical cancer is the fourth most common cancer in women. It causes 266,000 deaths worldwide in 2012, and accounts for 7.5% of all female cancer deaths [1]. Human papillomavirus (HPV) has been recognized as a necessary etiological agent of cervical cancer since virtually all cervical cancer specimens were positive for HPV [2]. Though majority of sexually active women have a risk to get HPV infection, most infections are naturally cleared within 2 years. Persistent infection may take 10 to 20 years to progress to precancerous lesions [3]. Even high-grade lesions may spontaneously regress and have less than 50% chance of progress to cancer [4]. Co-factors (listed in Tables 1 and 2), such as environmental carcinogens and cigarette smoking are believed to play a role contributing to HPV-associated carcinogenesis.

**Table 1:** International Agency for Research on Cancer classification of polycyclic aromatic hydrocarbons used in this study

Polycyclic Aromatic Hydrocarbons	IARC Group^a^
Acenaphthene	3
Acenaphthylene	N/A
Anthracene	3
Benz[a]anthracene	2B
Benzo[a]pyrene	1
Benzo[e]pyrene	3
Benzo[b]fluoranthene	2B
Benzo[ghi]perylene	3
Benzo[j]fluoranthene	2B
Benzo[k]fluoranthene	2B
Chrysene	2B
Dibenz[a,h]anthracene	2A
Fluoranthene	3
Fluorene	3
Indeno[1,2,3-cd]pyrene	2B
Phenanthrene	3
Pyrene	3

^a^IARC group 1, carcinogenic; group 2A, probably carcinogenic; group 2B, possibly carcinogenic; group 3, not classifiable; N/A, no information from IARC [5]. IARC, International Agency for Research on Cancer

**Table 2:** Co-factors contributing to HPV-mediated carcinogenesis recognised by IARC

Co-factors of HPV	References
Tobacco smoking	[30–33]
Hormonal contraception	[31, 34–36]
Number of pregnancies	[30, 37–39]
Nutrition/Dietary intake	[40–42]
Immunosuppression	[43–45]
Other infectious agent/inflammation	[46–49]

Polycyclic aromatic hydrocarbons (PAHs) are suspected to be one of these co-factors. PAHs are ubiquitous group of potent environmental pollutants that consist of 2 to 7 fused aromatic rings. PAHs have raised significant environmental concern because of their carcinogenicity, mutagenicity, and teratogenicity [5]. A group of 17 PAHs have been identified as priority pollutants by the Agency for Toxic Substances and Disease Registry (ATSDR) of the United States as they are suspected to be more harmful to humans.

Both epidemiological studies [6–9] and animal experiments [10–12] have suggested that PAH exposure can increase risk of various cancer types, e.g. skin, lung, bladder, upon PAH exposure. These PAHs were therefore classified as carcinogens by the International Agency for Research on Cancer (IARC) (listed in Table 1). Among these PAHs, benzo[a]pyrene (BaP) is the best characterized carcinogen. It is metabolized via cytochrome P450 enzymes to intermediates or metabolites, which can then bind to DNA and form DNA adducts [13, 14]. Unrepaired DNA adducts can be an important initiator of carcinogenesis [15, 16].

The general public is inevitably exposed to PAHs. For instance, BaP is a major component of cigarette smoke condensate and is present at 8–25 ng per cigarette [17]. BaP metabolites were found at elevated levels in the cervical mucus of women smokers [18]. Furthermore, it has been demonstrated that BaP can interact with HPV. BaP can increase HPV titer in cervical cells, implicating that BaP can modulate HPV life cycle, and possibly has a potential to affect viral persistence and cancer progression [19]. On the other hand, high-risk HPV infection has been found to substantially increase the overall metabolism of BaP to a more carcinogenic form [20].

Although epidemiological evidence has clearly suggested an etiological role of PAHs in cervical carcinogenesis, it is difficult to ascribe the observed health effects to a specific PAH, as most individuals are exposed to a mixture of PAHs on a daily basis. Current research on PAHs is mostly concentrated on BaP, yet the role of other PAHs should not be neglected as they also exist ubiquitously in the environment. To date, there has been no systematic study to delineate the oncogenicity of different PAHs in association with a ubiquitous viral carcinogen, HPV. The aims of this study were to compare the carcinogenic potential of different PAHs as a co-factor in HPV-mediated carcinogenesis, and to investigate the role of these PAHs during different stages of HPV-mediated carcinogenesis.

## Methods

According to ATSDR, the general public are more likely to be exposed to a group of 17 PAHs which exhibit adverse health effects (Table 1). We therefore decided to investigate the potential of these 17 PAHs as a co-factor with high-risk HPV on the aspects of primary cell transformation, cell proliferation, cell motility and invasiveness. HPV16 was chosen as the representative type for being the most prevalent and having the strongest association with cancer.

### Preparation of PAH stock solutions

The 17 PAHs were purchased from Sigma-Aldrich (St. Louis, MO, USA). Stock solutions of each PAH were dissolved in dimethyl sulphoxide (DMSO; Sigma-Aldrich) to a concentration of 10 μM.

### Cell lines

Human cervical cancer cell lines C33A (HPV-negative, mutant p53) and human breast cancer cell line MCF7 (HPV-negative, wild type p53) were generous gift from Prof. To K.F and Prof. Fung K.P from the Chinese University of Hong Kong, respectively. CaSki (HPV16-positive, wild-type p53, ATCC® CLR-1550) and SiHa (HPV16-positive, wild-type p53, ATCC® HTB-35) were purchased from the American Type Culture Collection (ATCC). These cells were maintained in Dulbecco’s Modified Eagle Medium (DMEM; Gibco, Waltham, MA, USA) supplemented with 10% fetal bovine serum (FBS; Gibco) at 37 °C in a humidified incubator with 5% CO_2_.

### Transformation assay

This assay was adopted from an in vitro co-transformation assay developed by Matlashewski [21]. The 9-day old Wistar Hannover rats were supplied by Laboratory Animal Service Centre (LASEC) of the Chinese University of Hong Kong. The rats were euthanized by CO_2_ suffocation prior to kidney extraction. Primary baby rat kidney (BRK) cells were extracted from kidneys. The carcass was disposed via LASEC. Primary baby rat kidney (BRK) cells extracted from kidneys were plated onto 10 cm dishes. Three micrograms of pcDNA16E7 and 1 μg of pEJ6.6 were co-transfected into BRK cells by DNA-calcium phosphate coprecipitation method. Plasmid pcDNA16E7 was constructed by cloning the HPV16 E7 gene, a major oncogene of the HPV genome, into the mammalian expression vector pcDNA3.1(+) (Invitrogen, Waltham, MA, USA) which contains a neomycin resistance gene for positive clone selection in subsequent experiments. Plasmid pEJ6.6 carries the H-ras oncogene and was a gift from Lawrence Banks (ICGEB, Italy). The H-ras oncogene was added in this system because HPV16 E7 gene alone was known to be insufficient to transform BRK cells [21]. After glycerol shock, the cells were selected in DMEM containing 10% FBS, 500 μg/mL Penicillin-Streptomycin-Glutamine (PSG; Gibco) and 220 μg/mL geneticin (Gibco), in the presence or absence of 1 μM PAHs. Eighteen days after transfection, the colonies were examined under microscope and scored according to the number as well as the size of colonies formed. Small, medium and large colonies were defined as < 6 mm^2^, 6–20 mm^2^ and > 20 mm^2^, respectively. Transformation score was calculated using the formula: number of large colonies × 8 + number of medium colonies × 4 + number of small colonies × 1. Colony formation index, defined as the score ratio of PAH to negative control, was used to compare the co-transforming ability of different PAHs.

### Cell proliferation assay

The effects of PAHs on cell proliferation was determined by WST-8 [2-(2-methoxy-4-nitrophenyl)-3-(4-nitrophenyl)-5-(2,4-disulfophenyl)-2H-tetrazolium] assay using a Cell Counting kit-8 (CCK-8; Dojindo, Shanghai, China). In brief, C33A and CaSki cells were seeded into 96-well plates at a density of 3 × 10^3^ cells/well in 100 μL of complete medium. The medium was replaced with complete medium containing 1 μM of PAH on the next day. After another 3 days, WST-8 (10 μL) was added to each well and incubated for 2 h at 37 °C. The optical density (OD) was measured at 450 nm by a VICTOR 3 Multilabel Plate Reader (PerkinElmer, Waltham, MA, USA). For long-term exposure, cells were treated with 1 μM of PAH for 3 months before analysis with cell proliferation assay.

### Wound healing assay

MCF7, C33A and SiHa cells were treated with 1 μM of PAH for three days prior to analysis by wound healing assay. Then 3 × 10^5^ MCF7 cells, 4 × 10^5^ C33A cells and 2 × 10^5^ SiHa cells were seeded into a 24-well plate so that cells would nearly reach confluency the next day. A wound was then introduced to the confluent cell monolayer by scratching with a 10-μL pipette tip. Cells were washed twice with phosphate-buffered saline (PBS) and monitored by time-lapse microscopy (Zeiss, Jena, Germany) for 48 h in DMEM supplemented with 2% FBS and 1 μM of PAH. Images were captured every hour. Cell migration index was calculated as the cell migrated area (T0 - T48) of experimental group divided by the control group in each cell line.

### Invasion assay

The effects of PAHs on invasiveness of MCF7, C33A and CaSki cells were assayed in 24-well Biocoat Matrigel invasion chambers (Corning, Corning, NY, USA) according to the manufacturer’s protocol. In brief, cells were treated with 1 μM of PAH for three days. Then, 5 × 10^4^ MCF7 cells, 1 × 10^5^ C33A cells and 5 × 10^4^ SiHa cells were resuspended in serum-free DMEM with 1 μM of PAH and seeded into the inserts. DMEM supplemented with 20% FBS was added to the lower chambers of the plate as a chemoattractant. After 24 h of incubation at 37 °C, the non-invasive cells were removed with a cotton swab. Cells that have migrated through the membrane to the lower surface of the insert were fixed with methanol and stained with hematoxylin. Invaded cells were counted under light microscopy. The relative invasion rate was represented by cell invasion index, which was calculated as the number of invaded PAH-treated cells divided by the number of invaded control cells. A test result would be considered as invalid when invaded cell number was less than 0.01% of the initial cell number, and its cell invasion index would be set as 1.

### Statistical analysis

Data were presented as mean ± SEM obtained from at least three independent repeats. The effects of PAHs on different assays were compared against controls by independent-samples T test. A *P*-value of less than or equal to 0.05 was considered as statistically significant.

## Results

### Effects of PAHs on HPV16E7-mediated cell transformation

**Fig. 1:**
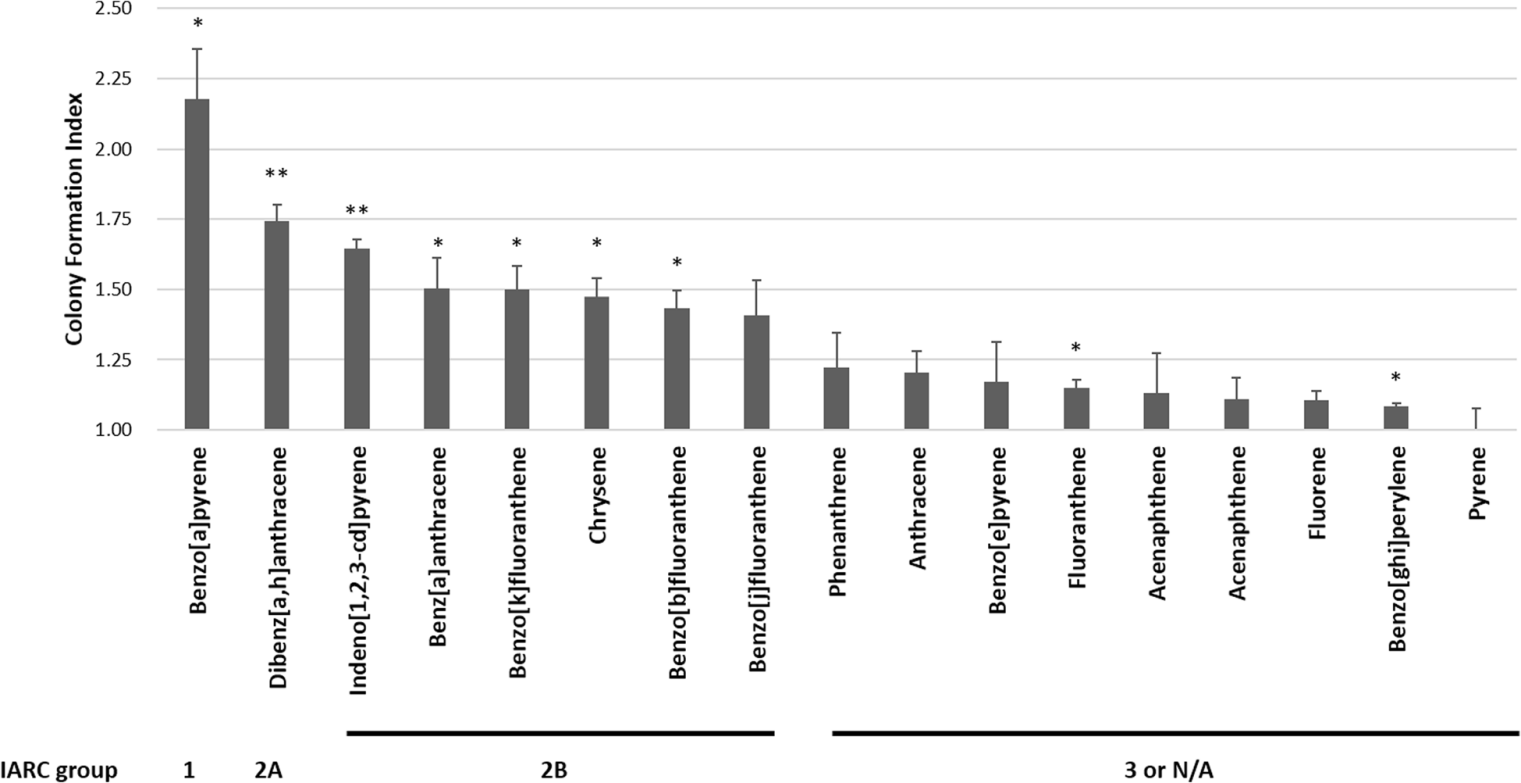
Effects of polycyclic aromatic hydrocarbons (PAHs) on HPV16E7-transfected baby rat kidney (BRK) cells. Cells transfected with pcDNA16E7 and pEJ6.6 were selected by 220 μg/mL of G418 in the presence of 1 μM of PAHs. Cells were fixed and stained on day 18 post-transfection. Transformed BRK cell colonies were divided into three size groups: small (< 6 mm^2^), assigned 1 point; medium (6–20 mm^2^), assigned 4 points; and large (> 20 mm^2^), assigned 8 points. The transformation score of each PAH was then calculated by the formula: number of small colonies × 1 + number of medium colonies × 4 + number of large colonies × 8. Co-transforming ability of PAH and HPV was represented by colony formation index (CFI = PAH transformation score / negative control transformation score). Data are the mean ± SEM values obtained from triplicate culture dishes per group from three independent experiments. Significant level: *p < 0.05, **p < 0.01, ***p < 0.001

Transforming ability of the 17 PAHs were tested using primary BRK cells. Our results showed a pattern coinciding with their IARC carcinogen classification (Fig. 1). Benzo[a]pyrene (BaP), one of the IARC group 1 carcinogens, displayed the greatest potential to enhance transforming ability of HPV16 E7 in primary cells (colony formation index = 2.2 ± 0.18, *p* < 0.05). Dibenz[a,h]anthracene (DBA), an IARC group 2A carcinogen, ranked the second (colony formation index = 1.7 ± 0.06, *p* < 0.01), and indeno[1,2,3-cd]pyrene (IDP) from IARC group 2B ranked the third (colony formation index = 1.6 ± 0.03, p < 0.01). Benz[a]anthracene, benzo[k]fluoranthene, chrysene, benzo[b]fluoranthene and benzo[j]fluoranthene ranking the fourth to the eighth, respectively, were also IARC group 2B carcinogens while the rest were from group 3 or not classified.

**Fig. 2:**
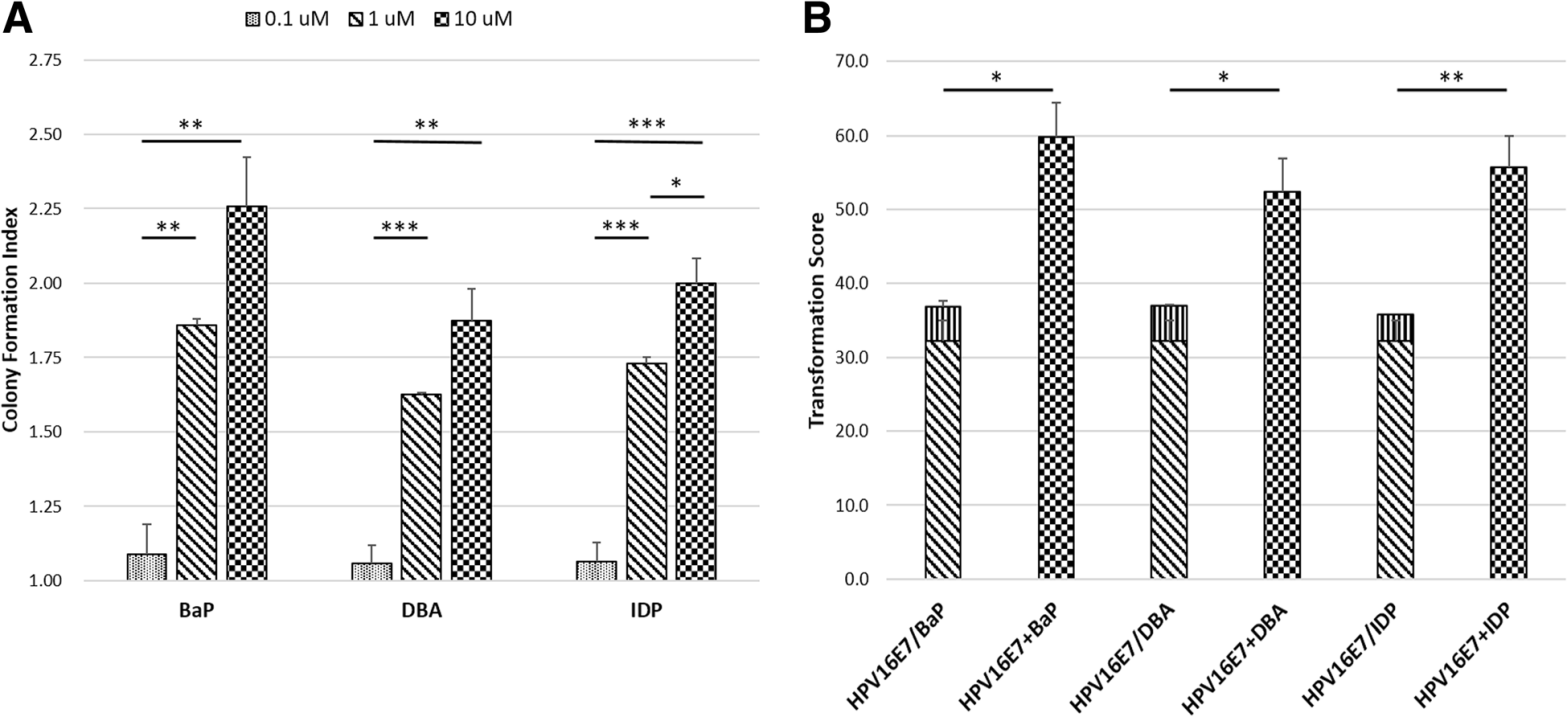
Dose-dependent effect and synergism of potent PAHs on HPV16E7-transfected BRK cells. Cells were treated with 0.1 μM, 1 μM and 10 of PAHs (**a**). The degree of dose-response was represented by colony formation index. Synergism of potent PAHs and HPV16E7 on BRK cell transformation (**b**), cells were treated with HPV16E7 alone; or, 1 μM of PAH alone; or, HPV16E7 together with 1 μM of PAH. Data are the mean ± SEM values obtained from triplicate culture plates per group from three independent experiments. Significant level: *p < 0.05, **p < 0.01, ***p < 0.001. BaP, benzo[a]pyrene; DBA, dibenz[a,h]anthracene; IDP, indeno[1,2,3-cd]pyrene

Among the 17 PAHs, 3 PAHs that has a higher ability to induce colony formation, namely BaP, DBA and IDP, with colony formation index of exceeding 1.5, were chosen to test if they can induce colony formation in a dose-dependent manner. The cells were treated with these PAHs at concentrations of 0.1 μM, 1 μM and 10 μM. Strikingly, our results showed that all three PAHs exerted profound dose-dependent effect in promoting colony forming ability of the HPV16E7-transfected BRK cells (Fig. 2a). Synergistic effect of PAHs with HPV16E7 was tested by treating BRK cells with HPV16E7 alone, PAH alone, or HPV16E7 together with PAH (Fig. 2b). Very few colonies (less than 5) appeared in PAH-only treated groups and they scored around 4 points (BaP-only: 4.7 ± 0.8, DBA-only: 4.8 ± 1.3, IDP-only: 3.6 ± 0.1). The HPV16E7-only group scored 32.2 ± 2.8 points. Interestingly, all three HPV16E7-PAH groups scored over 50 points (HPV16E7-BaP: 59.8 ± 4.7, HPV16E7-DBA: 52.3 ± 4.5, HPV16E7-IDP: 55.7 ± 4.3), and were significantly higher than the sum of HPV16E7-only group and their respective corresponding PAH-only group. These data indicated that PAHs exert synergistic effect rather than additive effect with HPV16E7 oncoprotein mediated transformation.

### Effects of PAHs on HPV16-mediated cell proliferation

**Fig. 3:**
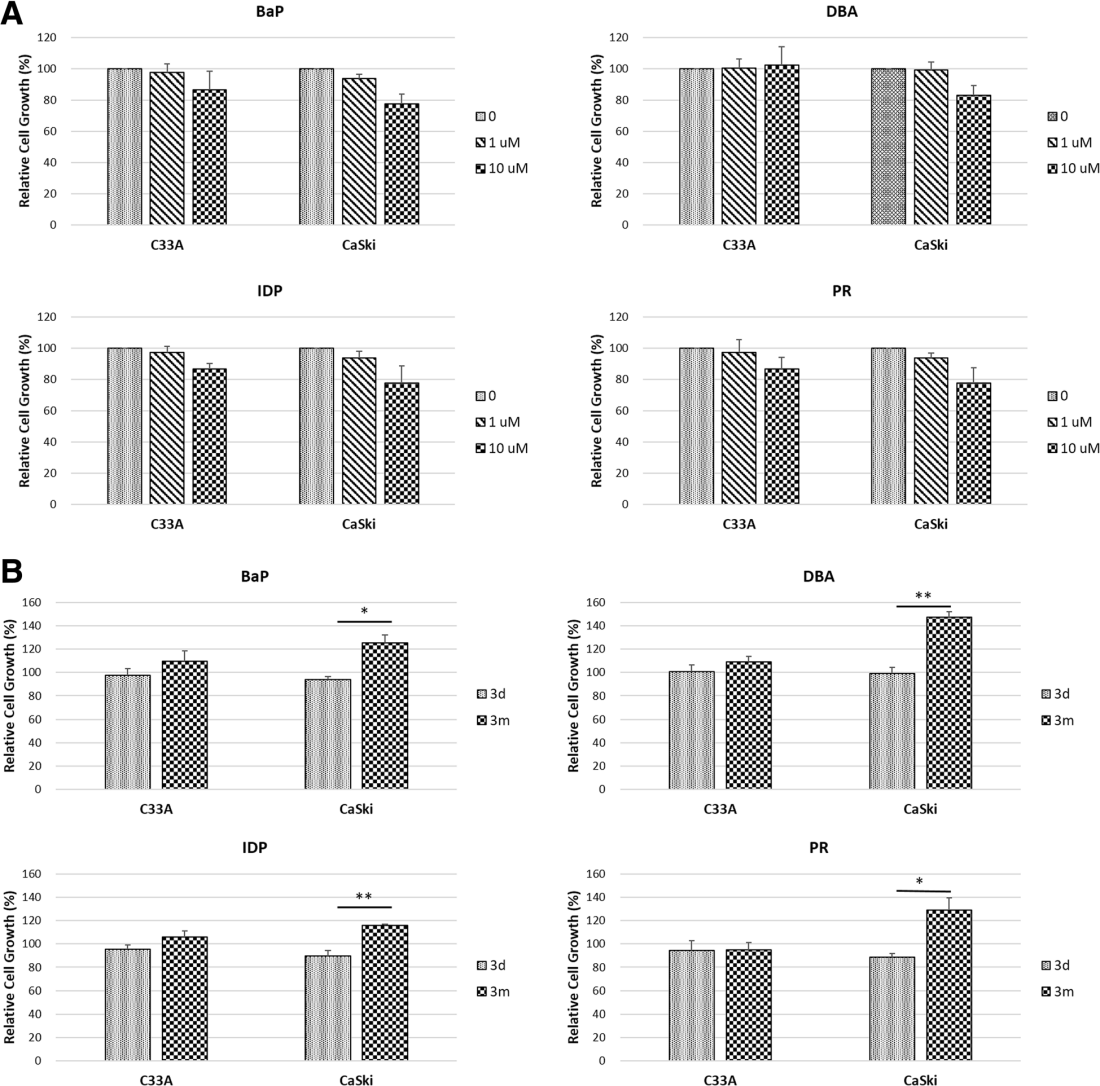
Short-term and long-term effects of PAHs on C33A and CaSki cell proliferation. Cells were treated with various concentrations (0–10 μM) of PAHs for three days (**a**). Cell proliferation was assessed by CCK-8 and represented as a percentage over DMSO-treated control group. Effects of PAHs were compared between three-day and three-month exposure of 1 μM of PAHs (**b**). Data are the mean ± SEM values obtained from triplicate culture wells per group from three independent experiments. Significant level: *p < 0.05, **p < 0.01, ***p < 0.001. BaP, benzo[a]pyrene; DBA, dibenz[a,h]anthracene; IDP, indeno[1,2,3-cd]pyrene; PR, pyrene

To investigate the effects of PAHs on HPV-mediated cell proliferation, we performed CCK-8 assay on two cervical cancer cell lines C33A and CaSki. C33A cells are HPV-negative, while CaSki cells contain integrated HPV16 genome. Four PAHs were selected for this and the subsequent experiments based on the results obtained from the transformation assay: BaP, DBA and IDP were the three most potential co-transforming compounds; pyrene (PR) showed low co-transforming ability and was used as a reference. The cells were treated with either 1 μM (low dose) or 10 μM (high dose) of PAHs for three days. As shown in Fig. 3a, all four PAHs, namely BaP, DBA, IDP and pyrene (PR), did not affect cell proliferation in both C33A and CaSki cells at 1 μM; while 10 μM of PAHs suppressed CaSki cell proliferation by 20% (*p* > 0.05). We therefore chose the non-toxic PAH concentration of 1 μM as the treatment dose in succeeding experiments.

Effects of long-term exposure of these PAHs were further examined. C33A and CaSki cells were treated with 1 μM of PAHs for three months and the relative cell growth was assessed by CCK-8. C33A cell proliferation was not significantly affected by any of these four PAHs. Significant increase in cell proliferation was observed in CaSki cells where BaP, IDP and PR led to around 1.2-fold (BaP: *p* < 0.05; IDP: *p* < 0.01; PR: p < 0.05), and DBA led to over 1.4-fold increment (p < 0.01) (Fig. 3b). These data suggested that short-term PAH exposure has little impact on proliferation of both HPV-positive and -negative cells, while long-term exposure could remarkably enhance cell proliferation in HPV-positive cell lines.

### Effects of PAHs on HPV16-mediated cell mobility

**Fig. 4:**
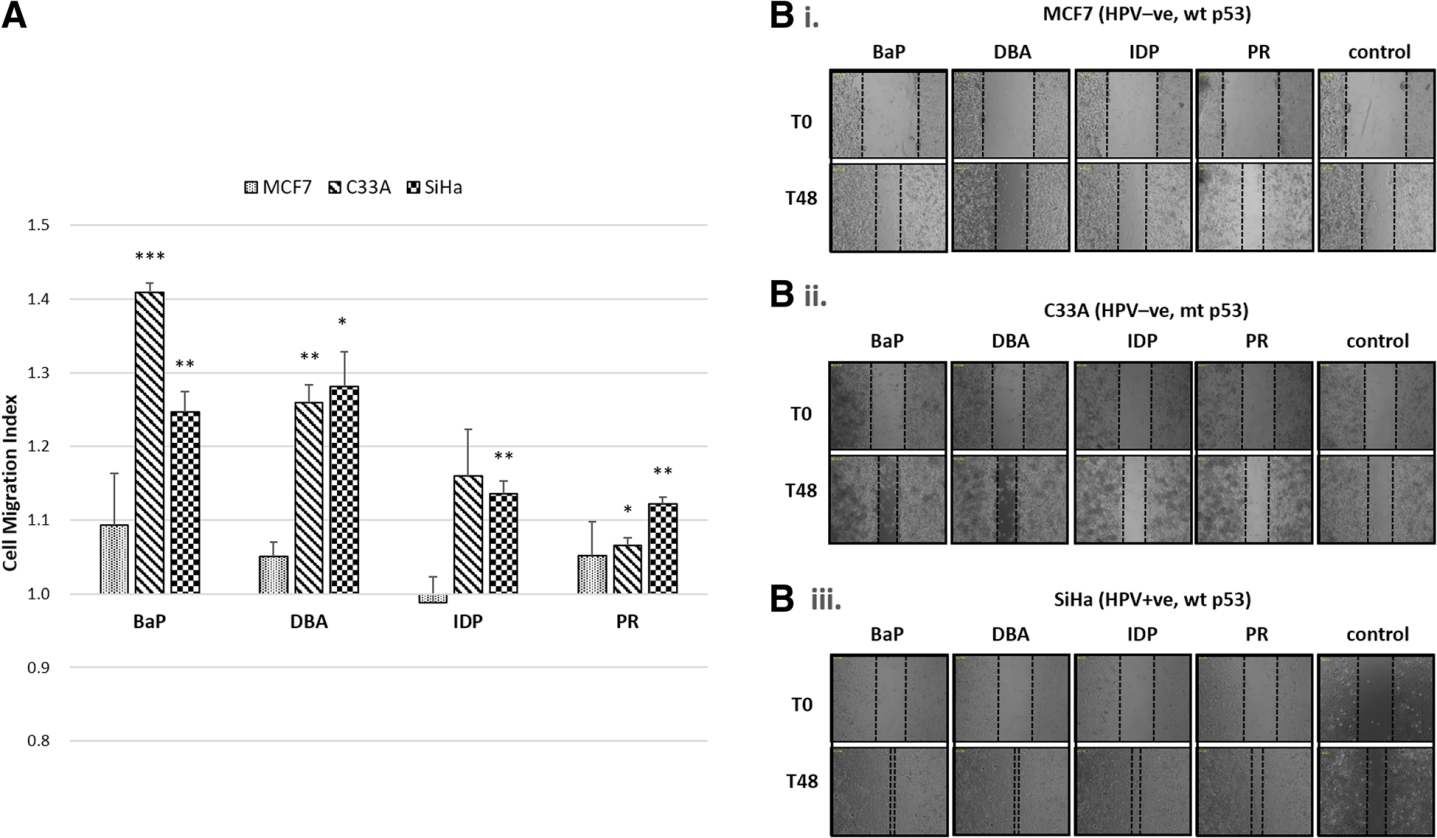
Effects of PAHs on HPV-infected cell motility. MCF7, C33A and SiHa cells were exposed to 1 μM of PAHs for three days. Changes of their migration capacity was assessed by wound-healing assay and represented by cell migration index (**a**). This index was calculated as the cell migrated area of experimental groups divided by the control groups in each cell line. Data are presented as the mean ± SEM from three independent experiments. Significant level: *p < 0.05, **p < 0.01, ***p < 0.001. Microscopic image captured at time point 0 h and 48 h are shown. Wounded area are indicated by dotted lines (**b**). BaP, benzo[a]pyrene; DBA, dibenz[a,h]anthracene; IDP, indeno[1,2,3-cd]pyrene; PR, pyrene

To examine the impact of PAHs on HPV-mediated cell motility, wound healing assay was performed. In this assay, instead of using CaSki, which has been known to possess migration and invasivion ability, grade II HPV16-positive SiHa cells were used. In addition, SiHa cells carry lower copy of integrated HPV16 genome than CaSki. We also included two HPV-negative cell lines with different p53 status, MCF7 and C33A cells. MCF7 mammary epithelial cells carry wild-type p53, and C33A cells carry mutant p53. Forty-eight hours after scratching, PAH-treated MCF7 cells showed similar migration capacity to the DMSO-treated control group (*p* > 0.05, data shown in Fig. 4). On the other hand, migration of both cervical cell lines were promoted by PAHs, especially by BaP and DBA. BaP induces increased migration capacity of C33A cells and SiHa cells by 1.4-fold (*p* < 0.005) and 1.25-fold (*p* < 0.01), respectively when comparing to their corresponding controls; and DBA induces increased migration capacity of C33A cells by 1.26-fold (p < 0.01), and SiHa cells by 1.28-fold (*p* < 0.05). Interestingly, migration of SiHa cells were slightly enhanced by the two less carcinogenic PAHs IDP (1.14-fold, p < 0.01) and PR (1.12-fold, p < 0.01) (Fig. 4). These results indicate that the more potent PAHs can lead to migration of cancer cells when comparing to the less potent PAHs. Exposure to potent PAHs can be a co-factor for cells harboring mutation on tumor suppressors, like p53, or expressing oncoproteins, like HPV-E6 and -E7, to migrate. In addition, PAHs may exert their carcinogenicity in a cell type-dependent manner, however, this is unclear and deserves further investigations.

### Effects of PAHs on HPV16-mediated cell invasiveness

**Fig. 5:**
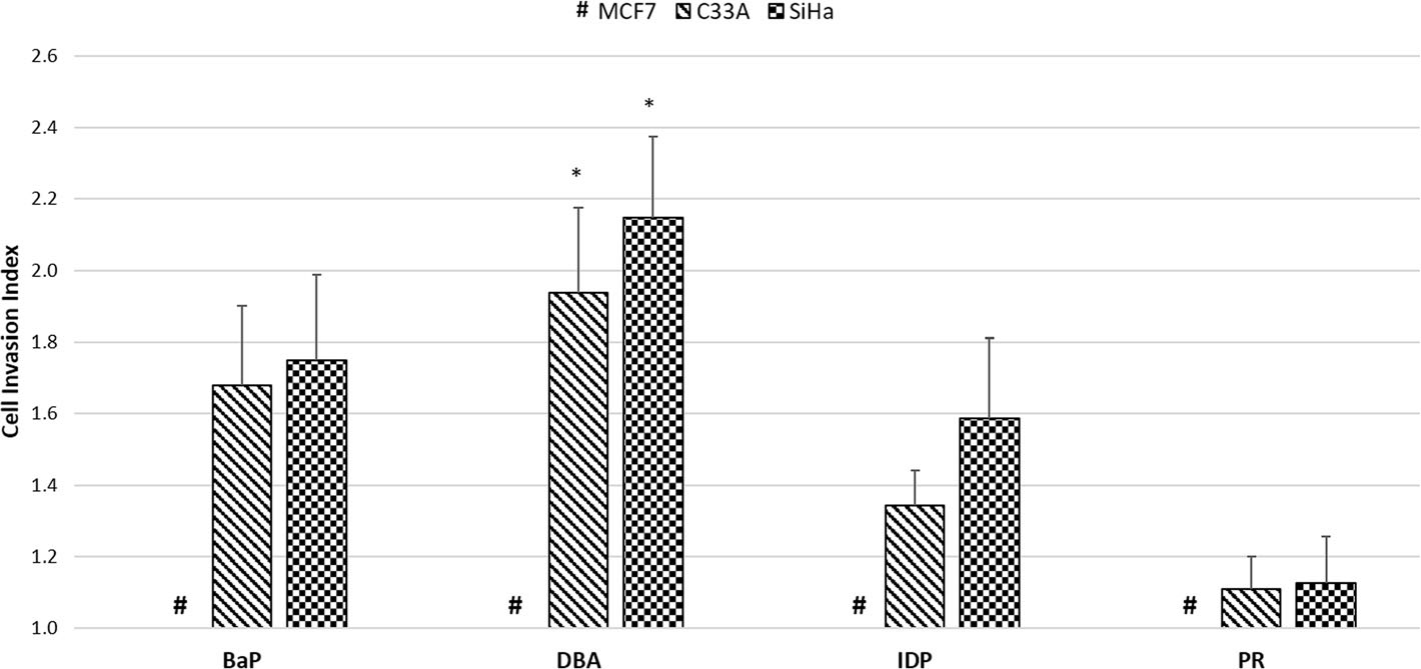
Effects of PAHs on HPV-infected cell invasiveness. MCF7, C33A and SiHa cells were exposed to 1 μM of PAHs for three days. Changes of their invasiveness was assessed by the transwell invasion assay and represented by cell invasion index. This index was calculated as the number of invaded cells from experimental groups divided by the control groups in each cell line. #, The invaded cell number of MCF7 was less than 0.01% of initial cell number, and the cell invasion index was set as 1. Data are presented as the mean ± SEM from three independent experiments. Significant level: *p < 0.05, **p < 0.01, ***p < 0.001. BaP, benzo[a]pyrene; DBA, dibenz[a,h]anthracene; IDP, indeno[1,2,3-cd]pyrene; PR, pyrene

The impact of four PAHs on HPV-mediated invasive capacity of MCF7, C33A and SiHa cells was also examined. Even in the presence of PAHs, MCF7 cells did not invade through the Transwell Matrigel-coated membrane and were therefore excluded from further analysis. Upon BaP, DBA, or IDP treatment, the invasive ability of C33A cells was increased by 1.68-fold, 1.94-fold (*p* < 0.05), and 1.34-fold respectively; while PR did not affect C33A cell invasiveness (*p* > 0.05, data shown in Fig. 5). Meanwhile all these four PAHs, namely BaP, DBA, IDP and PR boosted invasiveness of SiHa cells by 1.75-fold, 2.15-fold (p < 0.05), 1.59-fold and 1.13-fold, respectively (Fig. 5). Consistently, our results showed that PAHs can cooperate with HPV to substantially enhance cell invasion ability.

## Discussion

Since the 1970s, a close correlation has been observed between PAHs and the development of cancer. Epidemiological studies of occupational exposure, e.g. coke oven workers and aluminum smelter workers, showed clear excesses of lung, skin and bladder cancers; animal studies proved that certain PAHs are capable of inducing tumors [7, 22]. However, carcinogenic mechanism of PAHs remains elusive. The formation of DNA adducts is believed to be a key event. It has been suggested that the PAH-DNA adducts may generate mutations in ras proto-oncogenes and p53 tumor suppressor gene, which are highly involved in the tumorigenic process [5].

Winkelstein was the first to hypothesize that cervical cancer is strongly associated with PAHs in 1977 [23]. Nischan then correlated the relationship between PAH as a cofactor for cervical cancer development by a case-control study in 1988 [24]. Besides, exposure to traffic-related airborne PAHs was suspected to increase the prevalence of cervical dysplasia, a precursor lesion for cervical cancer [25]. Biochemical studies demonstrated that PAHs can reach and became metabolically activated in human cervix, resulting in DNA adducts. DNA adducts derived from BaP and other PAH compounds were detected throughout the cervix epithelium, indicating that PAHs may contribute to the etiology of cervical cancer in combination with HPV [18]. In a previous study, the tumor-initiating and tumor-promoting activities of three PAHs (benzo[a]anthracene, benzo[ghi]perylene and PR) were tested using BALB/c 3 T3 cells transfected with HPV16 E6 and E7 oncogenes [26]. It was found that benzo[a]anthracene, an IARC group 2B carcinogen, possessed both initiating and promoting activity; while benzo[ghi]perylene (IARC group 3) seemed to have tumor-promoting activity only and PR (IARC group 3) did not show any carcinogenic potential.

In this study, the effects of PAHs were examined in different stages of HPV16-mediated carcinogenesis: the BRK cell transformation assay represented tumor initiation, the CCK-8 assay was used for promotion, and the scratch assay as well as invasion assay were for progression. Our findings provide evidence that PAHs can enhance tumor-initiating ability of HPV16, and the effects observed are in line with the IARC classification. Our observations support that PAHs can contribute to HPV-mediated tumor promotion and progression. Of note, DBA (IARC group 2A), instead of BaP (IARC group 1), displayed the highest potential in augmenting proliferation, migration and invasion of HPV16-infected cells. Interestingly, PR (IARC group 3) showed no tumor-initiating ability but was found to be tumor-promoting in this study. Moreover, PR could increase migration of HPV16-infected cells, but not in HPV-null cells, even though it failed to stimulate invasion.

Two issues require careful consideration when relating our in vitro results to the in vivo system. First, the PAH level used in this study may be higher than the dose most people exposed to in daily life. Besides, PAH was directly applied to the target cells in this study, while in human body it reaches the target cell via circulation and diffusion through intervening tissues. For the in vivo study design, it is important to have the PAH level and exposure time to mimic the natural environment. Second, the in vitro monolayer cell culture and the in vivo cervical epithelia would have remarkable differences in response to carcinogens, as the former is lack of cell differentiation, three-dimensional cell-cell interaction, host immune surveillance and so on.

## Conclusions

Our findings suggest that both HPV infection and PAHs are critical factors in the development of cervical cancer. PAHs have a potential to co-operate with HPV to influence carcinogenesis at all three stages. Given the trend of increase in HPV-associated oropharyngeal cancers in many parts of the world [27], and higher chance of direct exposure of oropharyngeal cells to inhaled PAHs, further studies are needed to clarify the molecular mechanisms by which PAH exerts the oncogenic effects on HPV-infected cells which may lead to novel disease prevention and intervention. Pathways that can be mediated by both PAH and HPV, for example the epidermal growth factor and DNA repair pathways, are good candidates to start with [28, 29].

## Abbreviations

ATSDR: Agency for Toxic Substances and Disease Registry
BaP: Benzo[a]pyrene
BRK: Baby rat kidney
DBA: Dibenz[a,h]anthracene
DMSO: Dimethyl sulphoxide
FBS: Fetal bovine serum
HPV: human papillomavirus
IARC: International Agency for Research on Cancer
IDP: Indeno[1,2,3-cd]pyrene
PAH: Polycyclic aromatic hydrocarbon
PBS: Phosphate-buffered saline
PR: Pyrene
PSG: Penicillin-Streptomycin-Glutamine
